# Causation in Psychoanalysis

**DOI:** 10.3389/fpsyg.2013.00077

**Published:** 2013-02-25

**Authors:** Nikolai Axmacher

**Affiliations:** ^1^Department of Epileptology, University of BonnBonn, Germany; ^2^German Center for Neurodegenerative DiseasesBonn, Germany

**Keywords:** epistemology, philosophy of psychoanalysis, causal inference, hermeneutic explanations, intentionality

## Abstract

It has been argued that psychoanalytic and biological theories cannot be integrated because they rely on different epistemological grounds, namely on hermeneutic versus causal explanations, that are inconsistent with each other. Such inconsistency would seriously question the general possibility of neuropsychoanalytic research. Here, I review three important arguments that have been raised in favor of this inconsistency: first, that psychoanalytic attempts to overcome repression aim to go beyond causal relationships; second, that hermeneutic explanations are retrospective and context-dependent and therefore follow a different logic than causal explanations; and third, that only causal hypotheses are falsifiable, while the introspective reasons for one’s own behavior are not. I present arguments against each of these statements and show that actually, causal and hermeneutic explanations are, at least in principle, consistent with each other. The challenge for neuropsychoanalytic research remains to find indeed empirical examples of theories which are causal and hermeneutic at the same time.

## Introduction

Neuropsychoanalysis – the attempt to integrate psychoanalytic theory and practice with a consideration of the neural basis of human behavior, cognition, and affects – may take several forms. Initially, it referred to the psychoanalytic study and therapy of patients with brain lesions (Kaplan-Solms and Solms, 2000; Solms and Turnbull, 2002). Subsequent studies widened the scope by including experimental investigations of psychoanalytic concepts – from studies on the neural basis of psychodynamic therapy (e.g., Axmacher and Heinemann, 2012; Buchheim et al., 2012) to the operationalization of specific concepts such as the constancy principle (Carhart-Harris and Friston, 2010), dreams (e.g., Dresler et al., 2011; Ruby, 2011), repression (e.g., Anderson et al., 2004; see Axmacher et al., 2010 for a critique of current operationalizations), psychodynamic conflicts (Loughead et al., 2010), etc. In addition to this empirical research, the epistemological basis of combining psychoanalysis and neuroscience has been widely discussed. Here, my goal is to contribute to this discussion by focusing on one, particularly problematic aspect, namely the relationship of the hermeneutic (or “depth hermeneutic,” as it includes unconscious processes; Lorenzer, 1986) approach taken in the psychoanalytic attempt to understand and reconstruct conscious and unconscious narratives, and the scientific strife for explanations in terms of causal relationships. The following considerations do not intend to provide an exhaustive overview of all arguments raised on this issue. Instead, they aim to provide a limited personal account on a question which remains central for the neuropsychoanalytic endeavor.

## The Problem

During psychoanalytic therapy, analyst, and client aim to understand the analyst’s mental and affective life. Many aspects of this inner life appear initially absurd and paradoxical; the belief that even (and particularly) apparent nonsensical aspects are relevant and may in principle be understood is a cornerstone of psychoanalytic theory (Brenner, 1955). The process of understanding these phenomena has been conceptualized as “hermeneutic” – as a circular process by which an initial and superficial understanding is incrementally improved as analyst and client co-construct meaning through conscious and unconscious affective transference. Scientific researchers also search to understand seemingly random phenomena when they attempt to find regularities in their data. These regularities may then be used to generate predictions for future experiments and to build hypotheses about underlying causal laws. Freud throughout his life maintained the ideal to combine these two levels of investigation – to find scientific explanations for the conscious and unconscious psychic contents and somatic symptoms that he observed in his patients. In this combination of qualitative hermeneutics with quantitative theories, Freudian psychoanalysis had a unique dual epistemological character (Ricoeur, 1970). Many philosophers (e.g., Habermas, 2005) and psychoanalysts (e.g., Spence, 1982; Thomä and Kächele, 2006) criticized the biological ancestry of Freudian psychoanalysis and its attempt to find “metapsychological” laws of psychic life that resemble the explanations in the natural sciences; instead, they suggested to ground psychoanalysis on a purely hermeneutic basis.

However, purely hermeneutic explanations are epistemologically problematic because they typically act in a retroactive manner – they attempt to explain consisting affects, psychic contents, and somatic symptoms, but do not make predictions about future developments. Therefore, purely hermeneutic hypotheses are inherently difficult to falsify, which has been extensively criticized by philosophers such as Popper (1963). This problem would not occur if hermeneutic reconstructions were (at least in principle) consistent with causal explanations – in this case, one could predict that removal of the cause should alter its effect as well. Indeed, some philosophical accounts of psychoanalysis suggest that repressed conflicts generate neurotic symptoms in the same way as physical causes induce observable effects in the external world; in this case, one would predict that removal of repression during the course of psychoanalytic therapy should also alleviate the symptoms (e.g., Grünbaum, 1984).

On the other hand, several arguments suggest that hermeneutic reconstructions are fundamentally inconsistent with causal explanations. In the remainder of this article, I will discuss three such arguments and try to convince the reader that this apparent inconsistency does, in fact, not exist. The first argument is based on the introspective notion that we experience ourselves as free, whereas no freedom appears to exist in a causally closed world. Similarly, it has been stated that causal explanations are inconsistent with the therapeutic aim of an enhanced degree of autonomy. Second, psychoanalytic reconstructions attempt to provide conscious or unconscious *reasons* – for an action, a somatic symptom, or a psychic content such as an affect (for the opposite view that explanations of seemingly irrational behavior are based on causes but not reasons, see Davidson, 1982). However, providing a reason appears to follow a different linguistic logic than finding a cause: typically, reasons are only given retrospectively, for example to justify some action. This occurs in a specific social context. Therefore, depending on the context, very different reasons may be given to justify the same action. In contrast, a cause should always lead to the same outcome. Third, while hypotheses on causal connections are falsifiable, introspectively perceived reasons for my own behavior appear not to be – I know best the reasons why I acted in a certain manner.

## Argument 1: Freedom and Causality

Freud has been abundantly criticized for his scientific orientation. One of the most famous proponents of this view, the philosopher Juergen Habermas, focused on the process of repression, which Habermas understands as the transition of a psychic content from the domain of (conscious) intentional relations into the world of (unconscious) causal laws: “In contrast to conscious motivations, the unconscious ones hereby acquire the driving, instinctual character of something that uncontrollably compels consciousness from outside it. […] They are twisted and diverted intentions that have been turned from conscious motives into causes and subjected communicative action to the causality of ‘natural’ conditions” (Habermas, 2005, p. 161). The therapeutic work on repressed memories and affects therefore aims to *overcome causality*: “Analytic insight, however, affects the causality of the unconscious as such. Psychoanalytic therapy is not based, like somatic medicine, which is ‘causal’ in its narrower sense, on making use of known causal connections. Rather, it owes its efficacy to overcoming causal connections themselves” (p. 172).

Habermas understands the interpretation of the client’s life history during psychoanalytic therapy as a process of emancipation, which is fundamentally inconsistent with the causal explanation of symptoms in neurobiology. This fundamental dichotomy between causal and hermeneutic explanations is based on normative views of these two approaches: Habermas relates causal explanations to a suppressive, dominating stance toward (external and internal) natural processes, while hermeneutics are conceptualized as a communicative process governed by the ideal of the “unforced force of the better argument”. However, it remains unclear how the therapeutic transition from causal laws to hermeneutic relationships should occur. In fact, it seems inconsistent to posit on the one hand that psychopathological symptoms are caused by repressed conflicts, but that the revelation of these conflicts removes not only the cause, but the entire causal relationship (for this criticism, see Grünbaum, 1984). Furthermore, the claimed inconsistency between freedom and embedding in causal relationship is only apparent: through a thorough analysis of what we mean by the term “free,” philosophers such as Bieri (2001) demonstrated that actions are not perceived as free if they are unaffected by *any* causes (then they would rather be perceived as *random*), but if they are unaffected by external force and instead governed by good reasons. Similarly, psychotherapy should not aim to avoid *any* influences on the patient’s behavior, but rather help the patient to act according to influences he identifies with. In this respect, autonomy means to be governed by the *right* laws.

## Argument 2: The Linguistic Logic of Causal and Hermeneutic Explanations

While causal explanations state an inevitable and lawful relationship between cause and effect, hermeneutic explanations rely on deferred reconstructions. To assess whether this difference indeed leads to an inconsistency of these two types of explanations, the specific “inevitability” of causal explanations need to be considered in greater detail. Several concepts of causality exist. Scientific research depends on the investigation of necessary, lawful relationships. Such a necessary relationship corresponds logically to the modus ponens, i.e., to the formulation that in all cases of occurrence of a cause, the causally related effect occurs as well; in other words, that it is impossible that a cause may be observed without its causally related effect: a cause is a *sufficient* condition for an effect. It should be noted that according to the modus ponens, this relationship is not reversible, i.e., it is well possible that an effect occurs without the corresponding cause. Causes are not *necessary* conditions, because an effect may be well caused by different causes. Furthermore, predictions from causes to effects are usually only valid if other accompanying circumstances remain constant (“ceteris paribus”). These other circumstances can be considered additional causes for this effect – most effects are due to a multitude of causes (multicausality).

This description is actually well consistent with the deferred statement of reasons in hermeneutic explanations. Causes only need to induce specific effects inevitably – and this is indeed the case whenever reasons are accepted as valid explanations for actions. When I ask someone “Why didn’t you come to work yesterday?” and he responds “Because I was sick,” then his staying at home is considered as an inevitable effect of his disease. (Again, this explanation is only valid given other circumstances, *ceteris paribus* – e.g., that everybody who is sick stays at home.) Furthermore, this criterion exactly describes a characteristic feature of psychoanalytic interpretations. To be successful, these interpretations need to be consistent descriptions of the patient’s inner narrative, such that current symptoms appear as necessary consequences of their past. On the other hand, it is not claimed that this reconstruction is the only correct one; in principle, the current symptom may as well have been caused by other circumstances. Therefore, psychoanalytic interpretations give sufficient, but not necessary explanations of psychopathological symptoms, similar to causal explanations.

## Argument 3: Falsification and Surprise

Finally, a criterion for causal explanations is that cause and effect are only related by a mediating mechanism, so that this relationship does not exist *a priori*, or semantically. For example, one might say that growth of a tree (as an effect) is caused by water, sun light, and a cascade of biochemical processes (as causes), but not by the increased heights of the tree (which in this case is only the definition of “growth”). The relationship between cause and effect thus needs to be discovered as a “surprising” result of empirical observations. Therefore, hypotheses on causal relationships remain in principle falsifiable – their truth may be disputed, and additional evidence may be demanded. As hypotheses on causal explanations state a necessary, lawful relationship, they can be falsified even by a single observation of a situation in which a cause is not followed by an effect.

It has been suggested that these characteristics of hypotheses on causal relationships (to be surprising and falsifiable) do not apply for explanations of actions by a reason. According to this line of argumentation, a typical explanation from a first-person-perspective (e.g., “Why have you left the party?” – “Because it’s time for bed and I want to go home”) cannot be falsified: in these explanations, “[…] there is no question of ‘giving one’s evidence’ or of ‘making a mistake’ ” (Toulmin, 1954, p. 135). Now, one of the most important insights of psychoanalysis is that the transparency of the causes of one’s own behavior is only fictitious. For example, a patient suffering from alcohol dependence may indicate as a cause of his alcohol consume that he only drinks for pleasure; in reality, his alcohol consume may have an entirely different function, for example to interrupt unbearable thoughts and memories. Such rationalizations of behavior are typically not voluntary deceptions, but remain intransparent for the patient himself. This is not only the case during pathological behavior, but in general – the reasons for one’s own behavior are not necessarily correct and may actually be disputed and revised (“No, you did not go home because you were tired but because you couldn’t stand the people on that party!” – “Well, yes, you are right.”). The apparent transparency of conscious experience becomes problematic; oneself or others may provide more appropriate reasons for one’s behavior than initial introspection does.

Thus, Toulmin’s claim of a fundamental difference between intentional and causal explanations – that intentional explanations of one’s own behavior are undisputable and incorrigible – cannot be maintained. On the contrary: a thorough analysis of the criteria for successful interpretations in psychoanalytical therapies reveals that only those interpretations can be considered successful which are initially *surprising* for the patient. This has been described by the psychoanalyst Hamburger (1998) when he writes: “In the therapeutic setting, the psychoanalyst investigates causal relationships in a manner comparable to approaches in the natural sciences […]” (p. 269; translation, NA).

## Concluding Remarks

Taken together, these arguments show that causal and hermeneutic explanations are – in contrast to common belief – not only well compatible with each other, but even adhere to similar truth criteria. This, however, does not mean that current psychoanalytic theories are already both hermeneutic and causal; this would require a well-accepted metapsychological foundation, which allows one to actually generate falsifiable predictions. Indeed, a practical (rather than epistemological) difference between interpretations during psychoanalytic therapy and causal explanations in an experimental context is that in the latter case, a limited number of several possible causes is usually known in advance; during psychoanalysis, the situation is much more open and complex, making it more difficult to find possible explanations (apart from even testing them).

The epistemological consistency between causal and hermeneutic explanations is closely linked to the neuropsychoanalytic endeavor: on the one hand, it represents a crucial philosophical requirement for the overall possibility of this field; on the other hand, neuropsychoanalytic research may one day result in a new metapsychology, i.e., a psychoanalytic theory that is well-grounded in contemporary neuroscientific research. This has been described by the philosopher Achim Stephan: “By its new orientation on the cognitive sciences and neurosciences, psychoanalysis may even be strengthened: it would get rid of scientifically questionable ballast, but may also gain a truly convincing basis for some of its postulated processes. It is not at all impossible that adequate cognitive or neurophysiologic correlates of the different defense mechanisms can be found. […] The neurosciences and cognitive sciences may thus serve as a new and scientifically better supported basis for clinical theory than the Freudian metapsychology” (Stephan, 2002, p. 81; translation NA).

## Conflict of Interest Statement

The author declares that the research was conducted in the absence of any commercial or financial relationships that could be construed as a potential conflict of interest.
